# Structural flexibility of Toscana virus nucleoprotein in the presence of a single-chain camelid antibody

**DOI:** 10.1107/S2059798324000196

**Published:** 2024-01-24

**Authors:** Nicolas Papageorgiou, Amal Baklouti, Julie Lichière, Aline Desmyter, Bruno Canard, Bruno Coutard, François Ferron

**Affiliations:** a Université Aix-Marseille, Architecture et Fonction des Macromolécules Biologiques (AFMB)–UMR7257 CNRS, Case 925, 163 Avenue de Luminy, 13009 Marseille, France; b Unité des Virus Émergents (UVE: Aix-Marseille University–IRD 190–Inserm 1207), Marseille, France; c European Virus Bioinformatics Center, Leutragraben 1, 07743 Jena, Germany; Station Biologique de Roscoff, France

**Keywords:** *Bunyavirales*, Toscana virus, nucleoprotein flexibility

## Abstract

*Phenuiviridae* nucleoprotein is the main structural and functional component of the viral cycle, protecting the viral RNA and mediating the essential replication/transcription processes. The nucleoprotein (N) binds the RNA using its globular core and polymerizes through the N-terminus, which is presented as a highly flexible arm, as demonstrated in this article. The nucleoprotein exists in an ‘open’ or a ‘closed’ conformation. In the case of the closed conformation the flexible N-terminal arm folds over the RNA-binding cleft, preventing RNA adsorption. In the open conformation the arm is extended in such a way that both RNA adsorption and N polymerization are possible. In this article, single-crystal X-ray diffraction and small-angle X-ray scattering were used to study the N protein of Toscana virus complexed with a single-chain camelid antibody (VHH) and it is shown that in the presence of the antibody the nucleoprotein is unable to achieve a functional assembly to form a ribonucleoprotein complex.

## Abstract 2

Structural rearrangement of the Toscana virus nucleoprotein is induced by a single-chain camelid antibody.

## 1. Introduction

Toscana virus (TOSV) is an arbovirus transmitted by sandflies that is responsible for causing febrile illness and marginally aseptic meningitis (Cusi *et al.*, 2010 ▸; Charrel *et al.*, 2005 ▸). It belongs to the *Phenuiviridae* family of the *Bunyavirales* order, which contains viruses with worldwide distribution that cause asymptomatic to severe infections in humans and animals. TOSV is an enveloped virus with a segmented tripartite RNA genome of ∼11 kb. The L and M segments are of negative polarity and code for the RNA-dependent polymerase L and the glycoprotein precursor, while the S segment has an ambisense polarity and codes for the nucleoprotein (N) and the nonstructural proteins (NSs) (Bishop, 1986 ▸; Giorgi *et al.*, 1991 ▸). *Bunyavirales* nucleoprotein (N) is the most abundant viral protein in both infected cells and virions, and is a necessary cofactor of the polymerase, ensuring its processivity; it is one of the main structural and multi-functional component of the viral cycle (Ferron *et al.*, 2017 ▸). Unlike the nucleoproteins of other viruses (Laude & Masters, 1995 ▸, Papageorgiou *et al.*, 2020 ▸), N is a single-domain protein. By coating the viral RNA (vRNA), N (i) protects the viral genome from degradation, (ii) avoids the formation of double-stranded RNA (dsRNA) between viral vRNAs of opposite polarity and (iii) compacts the RNA into a flexible ribonucleoprotein (RNP). By recruiting a number of host proteins, N is also responsible for generating significant interference in the transduction pathway, signalling cellular infection (Papageorgiou *et al.*, 2020 ▸). To date, observations have shown that the structure of all N proteins of segmented RNA viruses presents a globular core which generally contains an RNA-binding cleft in its centre. In spite of limited sequence similarity, the RNA-binding cleft is positively charged to accommodate and guide the negatively charged phosphate backbone of the RNA. In the RNP, the RNA-binding cleft is oriented towards the centre of the polymer, ensuring complete protection of the vRNA. Either a single N-terminal (*Phenuiviridae*), both N- and C-terminal (*Hantaviridae*, *Peribunyaviridae* and *Tospo­viridae*) or central (*Nairoviridae*) multimerization extensions protrude from the core domain, revealing a variety of modes of oligomerization (Papageorgiou *et al.*, 2020 ▸). In the case of *Phenuiviridae* (Rift Valley fever virus and TOSV) the nucleoprotein mediates multimerization using an extended N-terminal arm (Le May *et al.*, 2005 ▸) that packs around the surface of the core domain of neighbouring protomers opposite the RNA-binding cleft (Ferron *et al.*, 2011 ▸). Several studies have shown that this arm is flexible, allowing complete closure of the RNA-binding cleft to different conformations in the multimer and allowing the core domain to accommodate the physical constraints of the RNP. This flexibility depends on the experimental conditions. In previous studies on the N protein using negative-stain transmission electron microscopy (TEM) it was shown that the N protomers form various types of multifold oligomers featuring tetramers, pentamers and hexamers assembled in open as well as closed conformations. This trend was observed in the case of Rift Valley fever virus (RVFV; Raymond *et al.*, 2010 ▸; Ferron *et al.*, 2011 ▸), as well as in the case of TOSV (Baklouti *et al.*, 2017 ▸). This structural organization is in agreement with the proposed formation of a helical model of the RNP (Olal *et al.*, 2014 ▸) and fits with the viral RNP particles that were originally observed by electron microscopy in the 1970s, showing circular flexible filamentous assemblies with no symmetry (Hewlett *et al.*, 1977 ▸). In the present study we have probed the assembly of N in the presence of a single-chain camelid antibody (VHH) targeting the N protein. We present structural results from single-crystal X-ray diffraction (XRD) and small-angle X-ray scattering (SAXS) of the N–VHH complex. The crystallo­graphic structure of the N–VHH complex shows that in the presence of VHH the N protein continues to form multimers within the crystal via its N-terminal region. However, the resulting multimer cannot accommodate RNA strands due to a complete inversion (‘flip’) between first-order N neighbours. This flipped N–N dimer is the basic element of the crystal organization.

In addition, we have observed that the N–VHH complex forms dimers as well as dimers of inverted dimers in solution. The inverted dimers observed in solution are in agreement with the structural organization observed in the crystal structure. Finally, enlightened by these results, we discuss the opportunities for the use of alternative binders such as nanobodies in a diagnostic or therapeutic approach.

## 2. Materials and methods

### 2.1. Protein production and purification

The production and purification of TOSV N under non­denaturing conditions have previously been described (Lantez *et al.*, 2011 ▸; Baklouti *et al.*, 2017 ▸). Briefly, after protein production in *Escherichia coli*, the bacterial pellets were resuspended in buffer consisting of 50 m*M* Tris pH 8.0, 10 m*M* imidazole, 0.1% Triton X-100, 5% glycerol, 0.25 mg ml^−1^ lysozyme, 1 m*M* phenylmethylsulfonyl fluoride and 2 µg ml^−1^ DNase I. In addition, 1 *M* NaCl and 3 *M* urea were added to the lysis buffer in order to remove nucleic acids from TOSV N. The protein was then purified as described previously under non-denaturing conditions (Lantez *et al.*, 2011 ▸) and stored in 10 m*M* HEPES pH 7.5, 300 m*M* NaCl.

### 2.2. Generation of VHH against TOSV N

A healthy llama (*Lama glama* from Ardèche Llamas, France) was immunized with 4 mg purified TOSV N produced as described previously and stored in 10 m*M* HEPES, 300 m*M* NaCl pH 7.5. During immunization, the protein was injected five times at one-week intervals. Lymphocytes were isolated from blood samples obtained five days after the last immunization. The cDNA was synthesized from purified total RNA by reverse transcription and was used as a template for PCR to amplify the sequences corresponding to the variable domains of the heavy-chain antibodies. The PCR fragments were then cloned into the phagemid vector pHEN4 (Lauwereys *et al.*, 1998 ▸) to create a VHH phage display library. Selection and screening of VHH were performed as described previously (Desmyter *et al.*, 2013 ▸).

### 2.3. Expression and purification of VHH

Selected nanobodies were cloned in a pHEN6 plasmid encoding an N-terminal pelB periplasmic signal sequence in frame with a VHH expression cassette and a C-terminal 6×His tag for detection and purification. Protein expression was achieved using WK6 bacteria cultivated in Turbo Broth medium supplemented with 100 µg ml^−1^ ampicillin and 0.1% glucose at 37°C until an OD_600 nm_ of 0.5–0.8 was reached. Expression was then induced by the addition of 1 m*M* isopropyl β-d-1-thiogalactopyranoside and growth was continued overnight at 25°C. The periplasmic proteins were extracted according to Skerra & Plückthun (1988 ▸). The bacteria were pelleted by centrifugation at 3500*g* for 15 min at 4°C. The pellet was resuspended in 9 ml cold TES buffer (0.2 *M* Tris–HCl pH 8.0, 0.5 m*M* EDTA, 0.5 *M* sucrose) per litre of culture and kept on ice for 1 h. The periplasmic proteins were removed by osmotic shock by the addition of 13.5 ml cold TES diluted four times with water. After 1–2 h on ice, the suspension was centrifuged at 21 700*g* for 30 min at 4°C. VHH was purified by immobilized metal-affinity chromatography (IMAC) purification. After loading samples for 1 h at 4°C, the resin was cleared of contaminants with wash buffer (50 m*M* phosphate buffer pH 8.0, 300 m*M* NaCl, 10% glycerol) and then eluted in elution buffer (50 m*M* phosphate buffer pH 8.0, 300 m*M* NaCl, 0.25 *M* imidazole). Fractions containing the purified nanobody were concentrated to 1 ml using Amicon Ultra-10K (Millipore) and dialysed in buffer consisting of 10 m*M* HEPES, 300 m*M* NaCl pH 7.5.

### 2.4. Purification of the TOSV N–VHH heterodimer

Monomeric fractions of N were pooled and complexed with purified VHH (ratio N:VHH = 1:1.5). After 1 h incubation at 4°C, size-exclusion chromatography (SEC) was applied to separate the N–VHH complex from free VHH using Superdex S200 16/60 (GE Healthcare) equilibrated with 10 m*M* Tris pH 8.0, 150 m*M* NaCl. The purified complex (N and VHH) was concentrated to 13 mg ml^−1^ and stored at 4°C.

### 2.5. Crystallization, X-ray diffraction, data collection and processing

Crystallization screening was performed by the hanging-drop vapour-diffusion method at 293 K using a nanodrop-dispensing robot (Mosquito, TTP Labtech). Optimal crystallization conditions were obtained by mixing 100 nl protein solution at 13 mg ml^−1^ with 100 nl reservoir solution consisting of 0.5 *M* MES pH 6.5, 25%(*w*/*v*) PEG 2000. The crystals were cooled in liquid nitrogen at 77 K; 10%(*v*/*v*) glycerol was used for cryoprotection. X-ray diffraction data were collected on the PROXIMA-1 beamline at the SOLEIL synchrotron (Coati *et al.*, 2017 ▸) using a PILATUS3 X 2M detector at a wavelength of 0.965 Å and a temperature of 100 K. The data were integrated using *XDS* (Kabsch, 2010 ▸), indexed in space group *P*4_1_2_1_2 (unit-cell parameters *a* = *b* = 54.7, *c* = 380.6 Å, α = β = γ = 90°), scaled and truncated to a resolution of 3.8 Å assuming a minimum acceptable signal-to-noise ratio of 2. The Matthews coefficient was determined to be 3.35 Å^3^ Da^−1^, suggesting a solvent content of 63%. Molecular replacement was performed using *Phaser* (McCoy *et al.*, 2007 ▸), and refinement and validation were performed using *Phenix* (Liebschner *et al.*, 2019 ▸). Due to the low resolution and because the occupancy and the thermal parameters (*B* factors) are highly correlated with one another, we refined occupancies as well as a common *B* factor for each amino acid (group *B* factors). Model building was performed and graphics representations were generated using *Coot* (Emsley *et al.*, 2010 ▸) and *UCSF Chimera* (Pettersen *et al.*, 2004 ▸) or *PyMOL* (version 2.0; Schrödinger).

### 2.6. Small-angle X-ray scattering (SAXS) measurements and data processing

SAXS profiles were measured on beamline BM29 at the European Synchrotron Radiation Facility (ESRF), Grenoble, France at an energy of 12.5 keV. The scattering wavevector *s* ranged from 0.025 to 5 nm^−1^. Data were recorded using a PILATUS 1M detector at a sample-to-detector distance of 2.43 m. Here, the scattering vector *s* is defined as

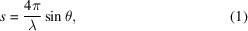

where λ is the wavelength of the incident radiation in nanometres and θ is half of the angle between the incident and scattered radiation. Five protein concentrations were measured: 0.7, 0.10, 0.13, 2.6 and 4.7 mg ml^−1^. The protein buffer consisted of 10 m*M* HEPES pH 7.5, 300 m*M* NaCl. All samples were centrifuged for 15 min at 15 000*g* before measurement in order to minimize contributions from aggregated particles. The measurement proceeded using 45 µl protein samples injected into a 1.8 mm capillary with flow to minimize radiation damage and data were collected at 277 K. Ten exposures of 1 s each were made for each protein concentration and were combined to give the average scattering curve for each measurement. The SAXS signal from the buffer was measured before and after measurement for each protein sample under the same conditions. The forward scattering intensity was calibrated using bovine serum albumin at 4.75 mg ml^−1^. Data were processed with the *ATSAS* package (Petoukhov & Svergun, 2007 ▸). The ten 1 s frames per protein were averaged using *PRIMUS* (Konarev *et al.*, 2003 ▸). Frames affected by radiation damage were excluded; the background due to the buffer was subtracted from the sample. The estimated correlation coefficient between the electron density and our model was estimated as follows: *UCSF Chimera* (Pettersen *et al.*, 2004 ▸) was used to create an electron-density map of our model using the molmap command. We then calculated the correlation coefficient for the fit of the two maps using *UCSF Chimera*.

## 3. Results and discussion

In the following section, we report and discuss details of our findings concerning the crystallographic structure of the N–VHH complex obtained by X-ray diffraction as well as by SAXS in solution.

### 3.1. Selection of the VHH used in the study

After the ligation of VHH coding sequence-specific PCR fragments into the pHEN4 phagemid, *E. coli* cells were transformed with the ligation reaction product to produce a library of clones that were quantified by colony-forming units. ∼3 × 10^7^ clones were obtained from three rounds of the panning phage display process. From the last round of panning, 47 individual clones were randomly picked, of which 31 produced a VHH with affinity for the antigen. Sequencing of the VHH coding sequences from the 31 candidates identified five different sequences. Large-scale production of the five VHHs led to the selection of two molecules that yielded quantities compatible with protein crystallization (>0.5 mg per litre of culture). The study describes and discusses the structural analysis of VHHs that led to a crystallographic complex with the antigen.

### 3.2. Crystallographic results for the N–VHH complex 

The crystallographic structure was obtained by molecular replacement (MR) using the core-domain structure of TOSV N (PDB entry 5fva; Baklouti *et al.*, 2017 ▸) and a dummy polyalanine VHH structure. After MR, the VHH structure was built according to the corresponding amino-acid sequence. A unique solution was found in space group *P*4_1_2_1_2 with two inverted N–VHH heterodimers per asymmetric unit.

**Figure 1:**
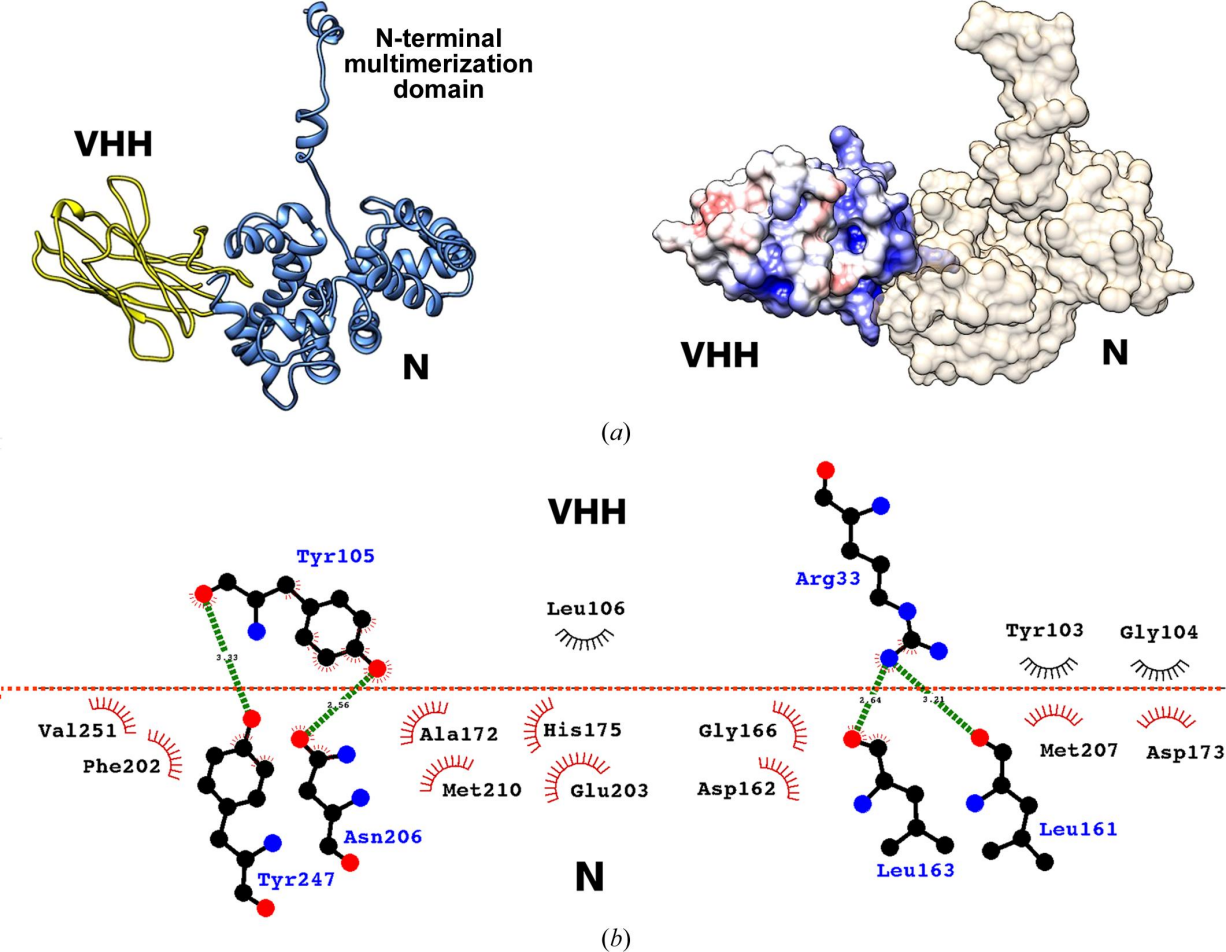
Crystal structure of the N–VHH complex. (*a*) The crystallographic structure of the complex in ribbon representation (left) as well as in surface representation. (*b*) Detailed representation of the residues participating in the N–VHH interaction as calculated by *DimPlot*. The upper part of the figure shows the VHH residues involved in the interaction, while the lower part shows the corresponding residues of the N protein. The red horizontal line symbolizes the interface between the two molecules. The interaction is mainly hydrophobic. The residues participating in this interaction are shown as semicircles, with the exception of the residues that form hydrogen bonds (shown as green lines). These residues are represented with their atomic structures, where C, N and O atoms are shown as black, blue and red circles, respectively.

Due to the relatively low resolution of 3.8 Å, only rigid-body refinement was applied. A first step of this refinement was applied to the core TOSV N domain and VHH structures. The refinement parameters were the occupancy and a common *B* factor for each amino acid obtained by choosing the ‘group *B* factors’ option in the *Phenix* refinement configuration. In a second step, we constructed the amino-terminal extension of TOSV N following the remaining electron density given by the refinement. An additional rigid-body refinement was applied to the ensemble of above structures, giving a final *R*_work_ of 0.31 and *R*_free_ of 0.36. Details of data collection and refinement are shown in Table 1 ▸. The structure has been deposited in the Protein Data Bank with PDB code 8rcq. Within the N–VHH complex the VHH is bound to the C-terminal subdomain of the globular protomer of TOSV N, while the N-terminal part of TOSV N protrudes and binds to the neighbouring inverted protomer in an inverted manner compared with the previously observed structures, as shown in Fig. 1 ▸ and discussed in detail below.

### 3.3. Unit-cell description and crystal-packing analysis

Within the entire unit cell, eight heterodimers (N–VHH complexes) are accommodated. Heterodimers (molecules *A* and *B* in Supplementary Fig. S1) polymerize in a linear fashion, forming fibres in the (*ab*) plane. Closely packed parallel fibres form flat layers. These planes are stacked along the *c* axis, from which the binding VHH protrudes, as mentioned previously. The layers are consolidated by the interaction of their protruding VHHs. In Supplementary Fig. S1, molecule *A* of the first layer interacts with the VHH of molecule *B* in the following layer, which is rotated by 25°. Layers along the crystallographic *c* axis are loosely connected and separated from one another by about ∼42 Å (Supplementary Fig. S1). In contrast, the crystal is much more compact within the plane defined by the *a* and *b* crystallo­graphic axes, with distances between crystal neighbours of less than ∼5 Å (Supplementary Fig. S1*d*). As a result, the crystal shows compact parallel planes loosely connected along the *c* direction. The existence of these long fibres as well as the large distance between the planes affects the overall order/stability of the crystal lattice along the *c* axis. This leads to a moderate diffracted intensity in the high-resolution region, resulting in the reported low-resolution structure (3.8 Å).

### 3.4. Description of a single N–VHH complex

In the case of a single N–VHH heterodimer, the N protein is in an ‘open’ conformation, with the flexible N-terminus extended away from the globular core of the N protein. The overall core is similar to the previously described structure; briefly, this is a bean-shaped structure composed of two all-α subdomains divided by a central cleft that accommodates the RNA. The C-terminal subdomain is the domain that interacts with the VHH (Fig. 1 ▸ *a*). The VHH is a β-sandwich composed of nine strands connected by flexible loops. The VHH binds to the N subdomain through an extended surface composed of several hydrophobic residues located in two side loops. Fig. 1 ▸(*b*) shows a detailed representation of the residues that participate in the interaction. The figure is divided into two parts separated by a red line symbolizing the N–VHH interface. The upper part shows VHH residues Tyr103, Gly104, Tyr105, Leu106 and Arg33. The lower part shows N residues Val251, Phe202, Tyr247, Asn206, Ala172, Met210, His175, Glu203, Asp162, Gly166, Leu163, Leu161, Met207 and Asp173. These residues were defined with the aid of *DimPlot*, which is a program within the *LigPlot Plus* suite for plotting protein–protein or domain–domain interactions (Laskowski & Swindells, 2011 ▸). On comparison with previously published structures of TOSV N (PDB entries 4csf and 5fva; Olal *et al.*, 2014 ▸; Baklouti *et al.*, 2017 ▸) no deformation was observed, confirming that binding of VHH does not alter the folding of N. The interface area between the two constituents of the complex (solvent-excluded surface area), as calculated using the PDB online facility *PDBePISA*, is ∼1400 Å^2^, which represents approximately 10% of the total accessible surface of the complex.

### 3.5. Description of the heterodimeric N–VHH complex

**Figure 2:**
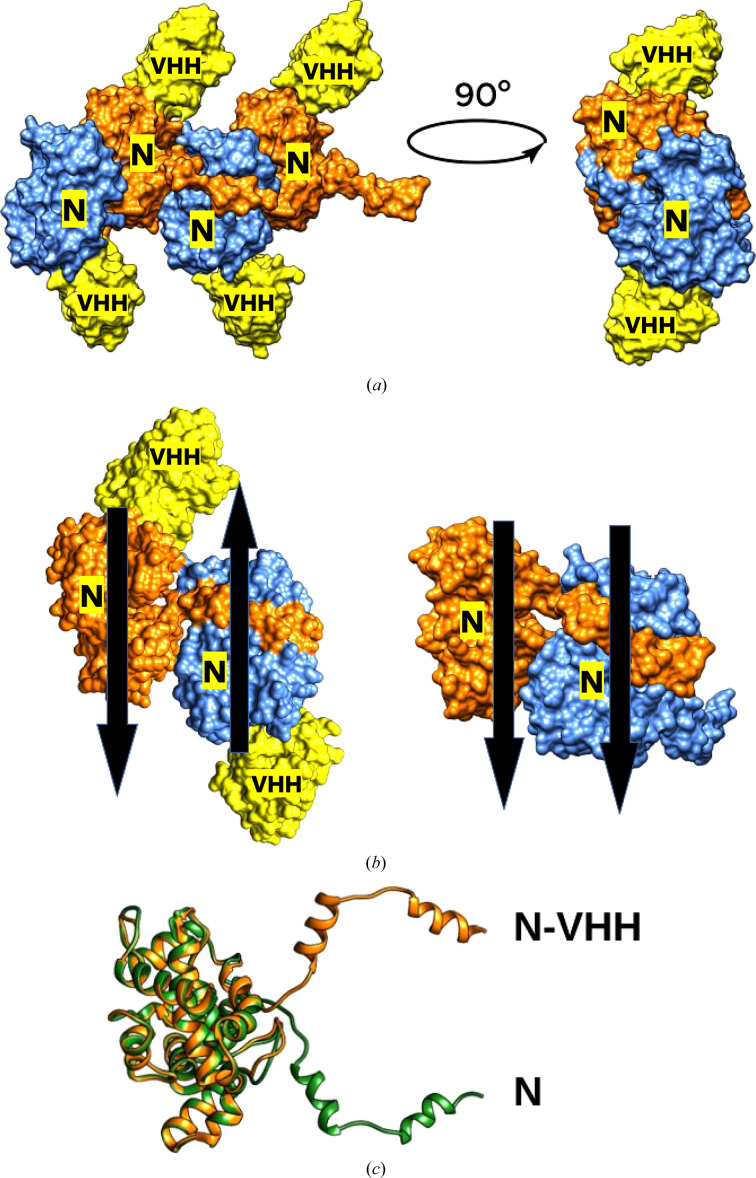
Comparison of the different assembly modes. (*a*) Four consecutive N–VHH complexes within the crystal are shown as coloured surfaces. The N protein of the complex is alternatively coloured blue and orange, while the VHH proteins are shown in yellow. (*b*) The complexes are aligned antiparallel to each other as marked by the black arrows. On the left, two N–VHH complexes are presented as described previously using the colour code described above. On the right, a molecular model of the nucleoprotein of Toscana virus (PDB entry 5fva) is presented. The black arrows show the orientation of the N proteins in both cases. (*c*) Superimposition of apo N (green) with N from the N–VHH structure (orange). Structures are shown in ribbon representation and show that when the cores are superimposed the N-terminal extension of N–VHH is flipped and distorted.

The assembly of single N–VHH complexes is different to the observed assembly of N protomers in the absence of VHH (Fig. 2 ▸). Indeed, in the absence of VHH the N promoters are oriented parallel to each other, as shown in the right part of Fig. 2 ▸(*b*). The black arrows exaggerate this trend visually. The first N neighbours are shown in blue and orange in this figure. The situation is strikingly different in the case where VHH is present. In this case the N protomers (blue and orange) are inverted with respect to each other.

**Figure 3:**
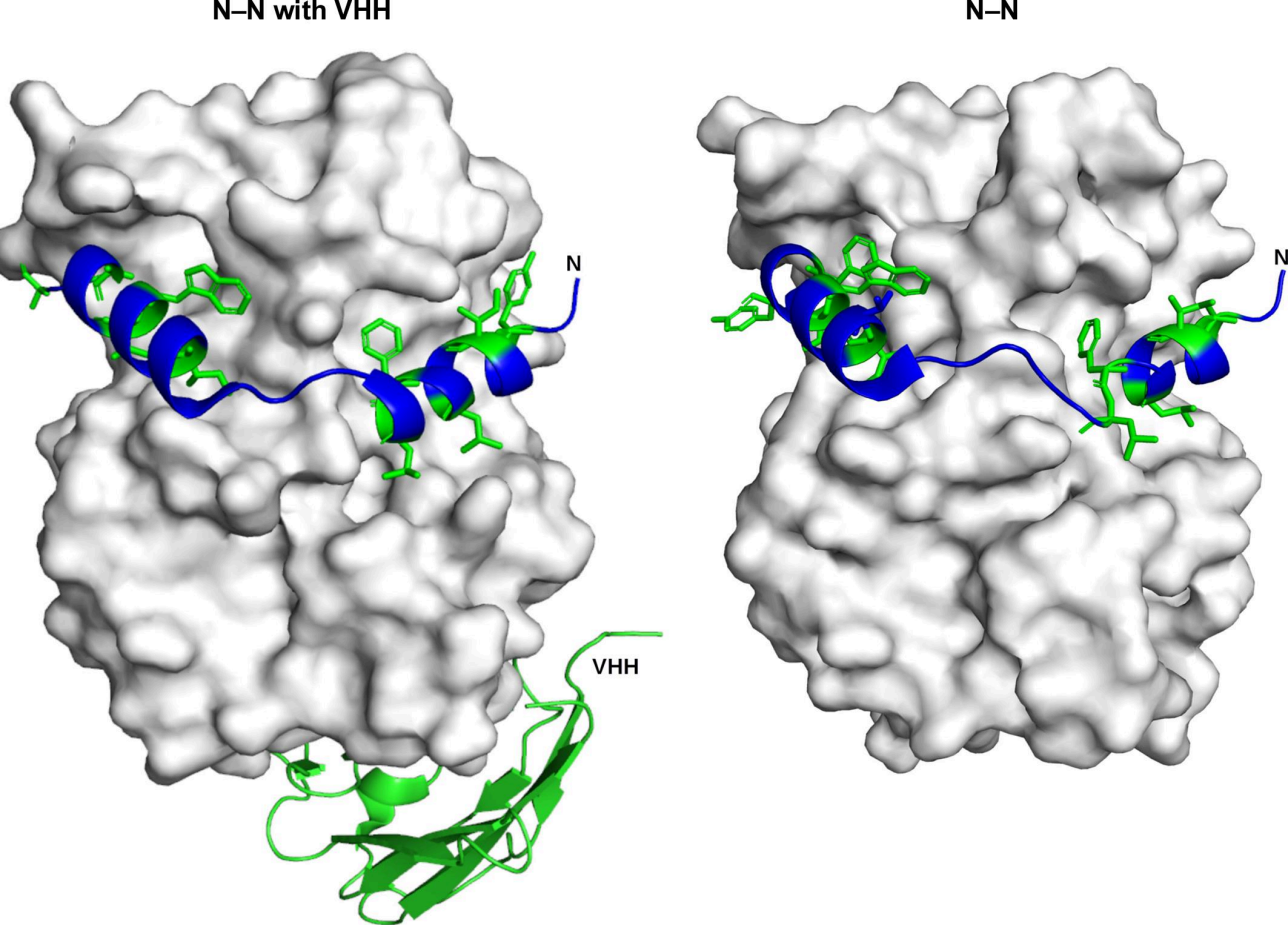
Enlargement of the interacting N-terminal arm of an adjacent protomer on the core of a neighbouring N of Toscana virus. The core is presented as a surface representation (white), while the N-terminal arm is shown as a ribbon representation (blue) and interacting amino acids are shown in stick representation (green). Left: the N–N interaction in the presence of the VHH. Right: the N–N interaction from PDB entry 5fva. It can be observed that the interaction involves the same binding mode and the same residues.

Despite this radical deformation, adjacent N promoters still remain attached by the same N-terminus as used in the absence of VHH (Fig. 3 ▸). Even under this extreme deformation, the mode of interaction between the N-terminal arm of an N protomer and the back of the adjacent protomer is not lost. There are two surprising observations: (i) multimerization is still possible despite the complete inversion of adjacent protomers and (ii) the amino acids involved in this multimerization in the N-terminal arm are the same hydrophobic residues (Fig. 3 ▸).

### 3.6. SAXS results for the N–VHH complex in solution

**Figure 4:**
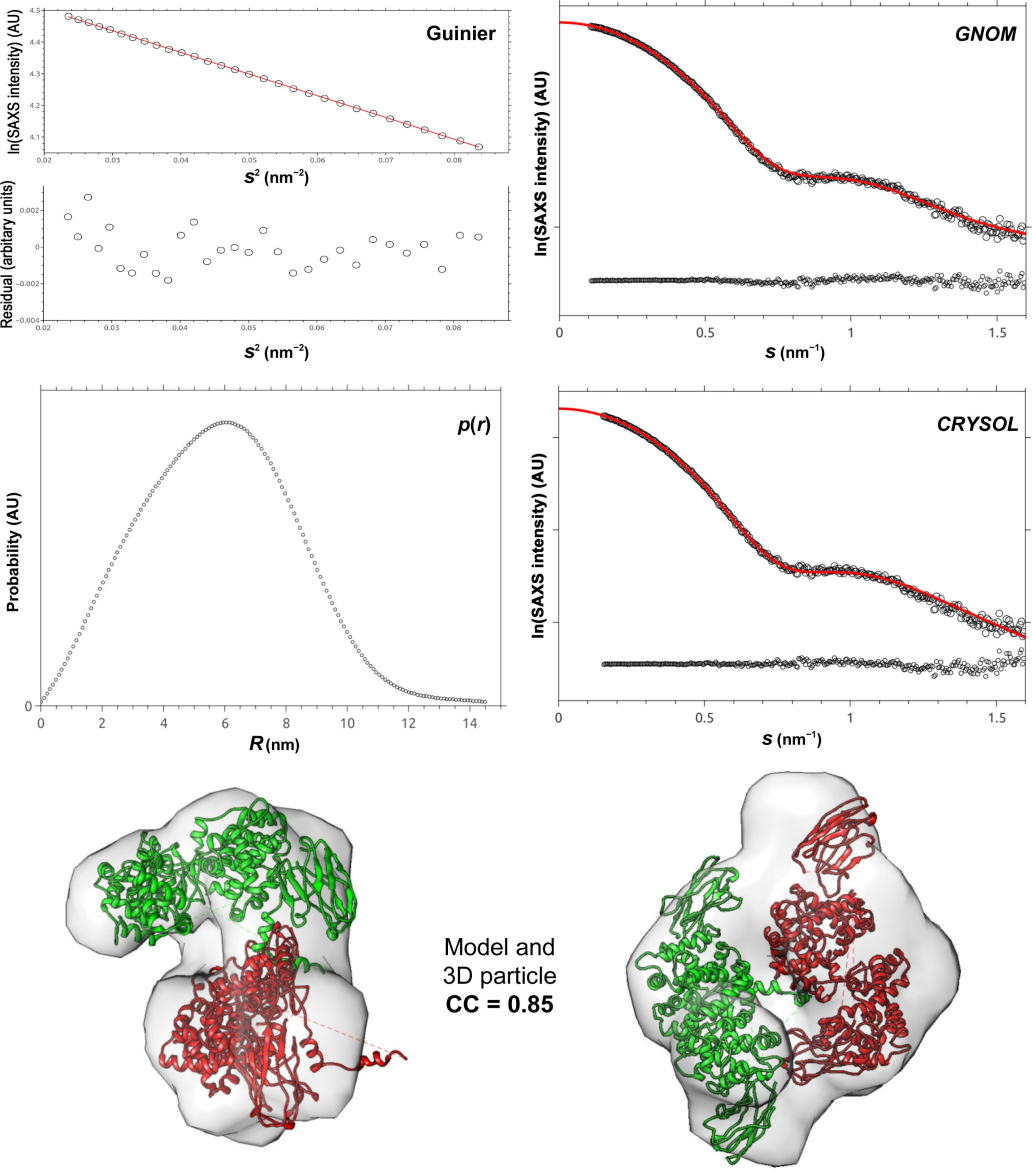
SAXS results for the N–VHH complex. The upper part of the figure shows the fit of the data within the Guinier region (*s*_min_ *R*_g_ < 0.65, *s*_max_ *R*_g_ = 1.3), the *GNOM* fit, the distance distribution *p*(*r*) and the *CRYSOL* fit between the data and the molecular model that we have elaborated in order to fit the data. In the *GNOM* analysis of the merged profile the data are shown as black dots and the *GNOM* fit as a red line. The error difference between the data and the *GNOM* fit is shown at the bottom of the figure with black dots. The distance distribution *p*(*r*) corresponds to the merged data calculated with a maximum particle dimension *D*_max_ of 14.6 nm (black dots). In the *CRYSOL* fit the error difference between the data and the molecular model is shown at the bottom of the figure with black dots. In the lower part of this figure we present a dimer of the N–VHH heterodimer fitted into the electron density calculated from the experimental data with *DAMMIN*. The correlation coefficient (CC) for this fit between the molecular model and the electron density is 0.85.

In order to obtain a deeper structural insight into the behaviour of the N–VHH complex assembly, we performed SAXS measurements in solution. We present our SAXS results in Fig. 4 ▸. The upper part of the figure shows the Guinier region, the *GNOM* fit, the distance distribution *p*(*r*) and the *CRYSOL* fit (Manalastas-Cantos *et al.*, 2021 ▸) between the data and the molecular model that we elaborated in order to fit the data. The Guinier region of the data was limited to between the typical limits of *s*_min_ *R*_g_ < 0.65 and *s*_max_ *R*_g_ = 1.3. The numerical linear fit of the data in the Guinier region is shown as a red line. Under the Guinier fit, the residual difference between the data and the fit is also shown. The good quality of the Guinier fit as well as the fact that the slope of the data was independent of the concentration in this region shows that there are no problems arising from aggregation or X-ray damage. From the ensemble of these data, we measured a mean value for the radius of gyration *R*_g_ of 4.5 ± 0.1 nm. The corresponding fit (data shown as black points and *GNOM* fit shown as a red line) was limited to within the typical region of *s*_max_(7/*R*_g_) = 1.62 nm^−1^ and *s*_min_(π/*D*_max_) = 0.25 nm^−1^, considering a maximum dimension of the particle *D*_max_ of 14.6 nm. The distance distribution *p*(*r*) converged smoothly to zero, confirming the good quality of the data as well as the correct choice of *R*_g_ and *D*_max_ parameters. Finally, we elaborated a molecular model based on the crystal structure discussed above. The *CRYSOL* fit between the data and this model is shown in the *CRYSOL* graph. The model fitted the data nicely within the *s*_max_–*s*_min_ region. The difference between the numerical fits and the data is shown as black points at the bottom of the *GNOM* and *CRYSOL* graphs, while the corresponding fit curves are shown as red lines. Finally, at the bottom of the figure we show the electron density calculated by *DAMMIN* (Svergun *et al.*, 1999 ▸) containing the molecular model proposed here. This model features four N–VHH complexes organized as two inverted heterodimers of N–VHH. The estimated correlation coefficient (CC) of the fit between the electron density and our model was 0.85. This observation is in agreement with the measured crystal structure discussed previously here.

### 3.7. Modelling of N–VHH in solution

In order to interpret our SAXS data, we considered the crystallographic N–VHH complex. To begin with, we estimated the mass of the particle using the empirical method proposed by Fischer *et al.* (2010 ▸). This method determines the molecular weight of proteins in dilute solution, with an error smaller than 10%, by using the experimental data of a single small-angle X-ray scattering (SAXS) curve measured on a relative scale. This procedure does not require the measurement of SAXS intensity on an absolute scale (independent of concentration) and does not involve comparison with another SAXS curve determined from a known standard protein. Using this method, we found the molecular weight of our particle to be 176.8 kDa. Taking into account the mass of a single N–VHH complex of 42.5 kDa, we estimated that the observed particle in this SAXS measurement comprises four N–VHH complexes corresponding to 4 × 42.5 = 170 kDa with an error of 4%.

In the following and in order to simplify the description of this complex structure, we will address the two inverted N–VHH complexes as a single entity. As the mass corresponds to four N–VHH complexes, we will deal with entity *A* and entity *B* from now on. Thus, we considered two separate entities (*A* and *B*) as presented in Supplementary Fig. S1(*c*) and placed them near to each other. Refinement of the relative positions of these entities was performed using *SASREF* (Petoukhov & Svergun, 2005 ▸). The resulting model fits the data and we believe that it is representative of the observed particle.

In the case of the crystal, entities *A* and *B* are parallel along the *c* axis, as shown in Supplementary Fig. S1(*c*). In the case of the SAXS model the two entities are no longer parallel but form an angle of about 30°. Due to the low resolution of the SAXS data, 1/*s*_min_ = 4 nm, we cannot conclude whether the two entities *A* and *B* in solution are connected via their N-terminal region or whether they are floating one near the other with no specific mechanism of connectivity. Previously, it has been shown that the N protein of phleboviruses can also present a closed conformation considered as an auto-inhibited conformation that prevents polymerization (Raymond *et al.*, 2010 ▸; Ferron *et al.*, 2011 ▸). In this state N remains monomeric. In all other structural studies, N was always associated in an oriented polymeric fashion as originally described by Ferron *et al.* (2011 ▸) and there has been no evidence, either from crystallography or in solution, that the inverted association, as presented in this study, exists naturally (Raymond *et al.*, 2010 ▸, 2012 ▸; Ferron *et al.*, 2011 ▸; Jiao *et al.*, 2013 ▸; Zhou *et al.*, 2013 ▸; Olal *et al.*, 2014 ▸; Baklouti *et al.*, 2017 ▸); we therefore conclude that the effect of a VHH on N dramatically affects its ability to form functional ribonucleoprotein and exclude the hypothesis that it could be a second auto-inhibited conformation.

Considering these results, one could conceive of the use of monoclonal antibodies (mAbs) as a therapeutic strategy or in a diagnostic context. Monoclonal antibodies, including VHH, have found extensive application in the clinical realm, playing a pivotal role in both diagnosis of and therapeutic interventions for various human disorders, including cancer, infectious diseases and immune-response modulation (Waldmann, 1991 ▸; Pelegrin *et al.*, 2015 ▸). In 2020, in the context of the COVID-19 pandemic, these mAbs therapies received emergency-use authorizations from the US Food and Drug Administration (FDA). However, the initial promise of these therapies was short-lived, as the emergence of variants posed a formidable challenge. *In vitro* neutralization tests indicated a diminished likelihood of mAbs therapies remaining efficacious against new variants (Kozlov, 2021 ▸). This observation underscores the dynamic nature of the therapeutic landscape and emphasizes the need for continuous adaptation in the face of evolving pathogens. To our knowledge there are no mAbs (or VHH) therapies currently in development against the nucleoprotein of TOSV or other viruses belonging to the *Phlebovirus* genus. However, in *Bandavirus*, a genus that also belongs to the *Phenuiviridae*, a recent study has identified two epitopes recognized by mAbs that could be used for diagnostics (Qian *et al.*, 2023 ▸). One of these epitopes would structurally overlap with that recognized in this study. However, the sequence of the target epitope is not conserved, even within *Phlebovirus* (Supplementary Fig. S2). This study reveals that the nucleoprotein possesses the capability to elicit an antibody response, suggesting that it could be used as a material for the development of viral antigen-detection methods against TOSV and probably other phleboviruses.

In conclusion, several observations have been made concerning the effect of a VHH on an N protein: (i) the amino-terminal region of the N protein is responsible for multimerization of the RNP and the extreme flexibility of the amino-terminus is critical for this process to be achieved, (ii) a significant perturbation in the assembly mode of N occurs in the presence of the VHH and (iii) within the N–VHH crystal the N protomers continue to polymerize, forming long fibres, which is incompatible with RNA encapsidation. As the presence of a VHH prevents N from assembling as a functional RNP, these results demonstrate that VHHs can be used as molecular probes for the identification of sequences or structures associated with viral functions that can be targeted for the rational development of antivirals.

## 4. Related literature

The following reference is cited in the supporting information for this article: Robert & Gouet (2014 ▸).

## Supplementary Material

PDB reference: Toscana virus nucleoprotein, 8rcq

Supplementary Figures. DOI: 10.1107/S2059798324000196/jc5062sup1.pdf

## Figures and Tables

**Table 1:** Data-collection and refinement statistics for the Toscana virus N–VHH complex Values in parentheses are for the highest resolution shell.

Wavelength (Å)	0.9801
Resolution range (Å)	47.46–3.80 (3.94–3.80)
Space group	*P*4_1_2_1_2
*a*, *b*, *c* (Å)	54.75, 54.75, 380.65
α, β, γ (°)	90, 90, 90
Total reflections	70195 (7334)
Unique reflections	6413 (637)
Multiplicity	10.9 (11.5)
Completeness (%)	99.47 (99.37)
Mean *I*/σ(*I*)	12.00 (1.60)
Average *B* factor (Å^2^)	159.71
*R*_merge_	0.20 (3.10)
*R*_meas_	0.21 (3.23)
*R*_p.i.m._	0.06 (0.94)
CC_1/2_	0.10 (0.78)
Reflections used in refinement	6393 (634)
Reflections used for *R*_free_	636 (64)
*R*_work_	0.31 (0.45)
*R*_free_	0.36 (0.49)
No. of non-H atoms	2763
Protein residues	366
R.m.s.d., bond lengths (°)	0.003
R.m.s.d., angles (°)	0.59
Ramachandran favoured (%)	94.66
Ramachandran allowed (%)	4.78
Ramachandran outliers (%)	0.56
Rotamer outliers (%)	0.00
Clashscore	6.70
