# Supplementary material for: Structural flexibility of Toscana virus nucleoprotein in the presence of a single-chain camelid antibody
Source: Acta Crystallogr D Struct Biol. 2024 Jan 24;80(Pt 2):113–22. doi: 10.1107/S2059798324000196 (PMC10836398; doi:10.1107/S2059798324000196)
Supplement: Supplementary file 1 [file d-80-00113-sup1.pdf]

STRUCTURAL BIOLOGY

Volume 80 (2024)

Supporting information for article:

Structural flexibility of Toscana virus nucleoprotein in the presence of a single-chain camelid antibody

Nicolas Papageorgiou, Amal Baklouti, Julie Lichière, Aline Desmyter, Bruno Canard, Bruno Coutard and François Ferron

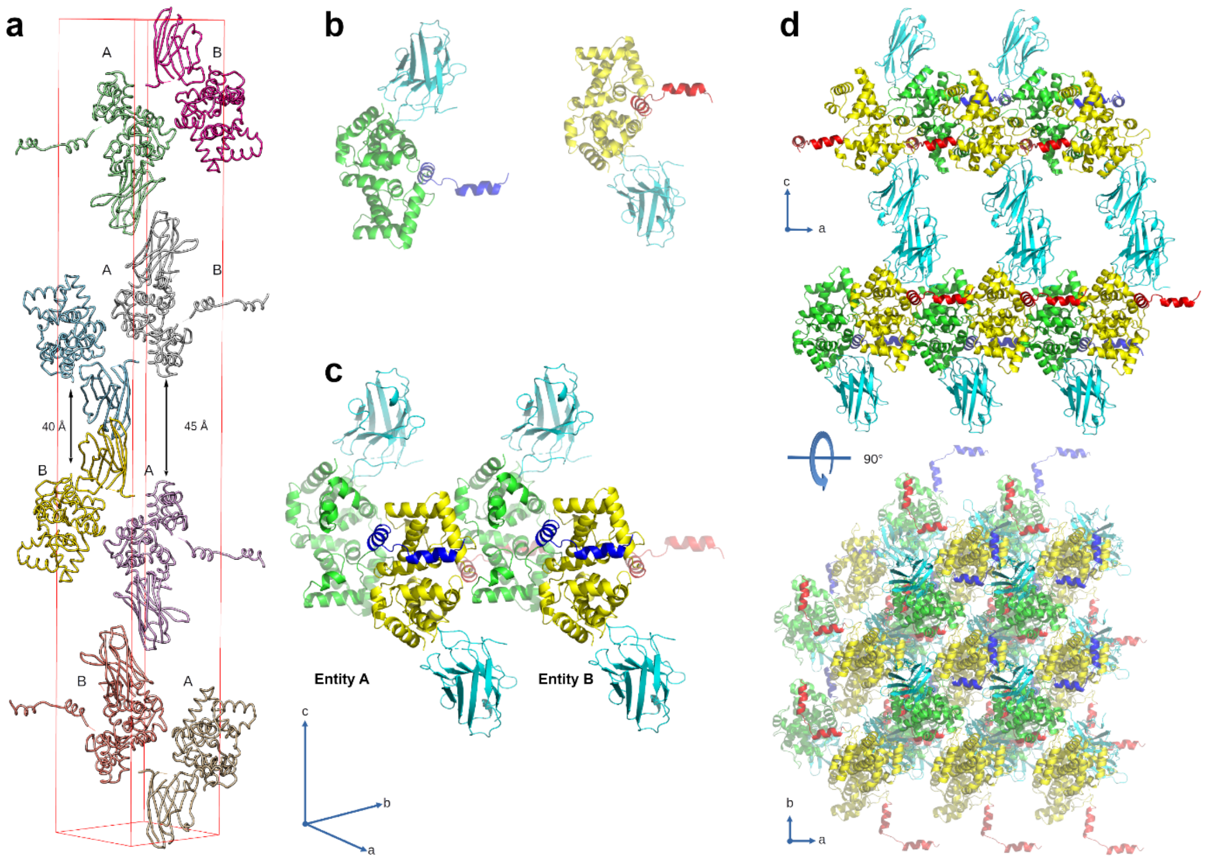

Figure S1 The unit cell of the Toscana virus N-VHH heterodimer and its different orientation within the unit cell. *(a)* One heterodimer is labelled A and the other B. *(b)* Dissociation of each heterodimer in shown in ribbon. N core of A is coloured in yellow and its N-terminal is in red,while N core of B is coloured in green and its N-terminal is in blue. The bound VHH on A and B is coloured in cyan. *(c)* Detail of 4 heterodimers named Entity A interacting with the neighbouring 4 heterodimers name Entity B. This assembly Entity A & B is the basic structural motif repeated along the a axis forming a fiber. *(d) Top* Representation of the crystal packing along a axis forming the fibers separated by two interacting VHH. *Bottom* Representation of the crystal packing at 90° showing a tight compact crystal.

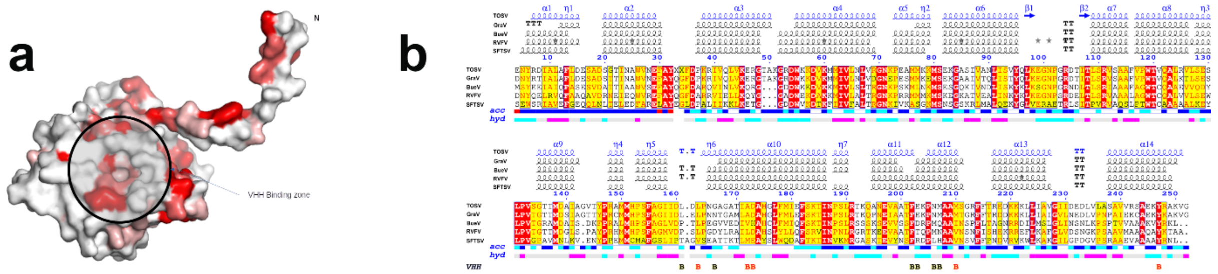

Figure S2 *(a)* Sequence conservation of N TOSV of the VHH binding site plotted on the surface. Conservation is plotted according to ENDSCIPT parameter (White color no conservation Red Identical). *(b)* Sequence alignment of Phleboviruses N sequences retrieved by ENDSCRIPT (Robert 2014)(default search parameter) using TOSV N-VHH structure as input search. Residues involved in binding are marked with B in red if the distance is less than 3.2 Å and in black if it is in the range 3.2-4.0 Å.
